# Clinicopathological Spectrum and Treatment Outcomes of Cryofibrinogen-Associated Nephropathies

**DOI:** 10.1016/j.ekir.2025.10.022

**Published:** 2025-11-05

**Authors:** Julien Dang, Stanislas Faguer, Noémie Jourde-Chiche, Vincent Javaugue, Frank Bridoux, Bénédice Puissant, Xavier Heim, Jean-Jacques Boffa, Hélène François, Yanis Tamzali, Evangéline Pillebout, Eric Daugas, Renato Monteiro, Laurent Daniel, Kévin Chevalier, Vincent Audard, Margaux Van Wynsberghe, Dominique Guerrot, Mathieu Legendre, Sébastien Sanges, Céline Lebas, Nans Florens, Charles Ronsin, Charlotte Mussini, Sophie Ferlicot, Sarah Mouawad, Renaud Snanoudj, Ana Pimentel, Marie Essig, Marc Pineton De Chambrun, Pascale Ghillani-Dalbin, Benjamin Terrier, Mohamad Zaidan

**Affiliations:** 1 Assistance Publique des Hôpitaux de Paris (AP-HP), Hôpital Ambroise Paré, Service de Néphrologie et Dialyse, Boulogne-Billancourt, France; 2 Université Versailles-Saint-Quentin, UFR Simone-Veil Santé, INSERM U1179, Montigny-le-Bretonneux, France; 3 AP-HP, Hôpital de Bicêtre, Service de Néphrologie et Transplantation, Le Kremlin-Bicêtre, France; 4 Hôpital de Rangueil, Service de Néphrologie et Transplantation d’Organes, Toulouse, France; 5 Aix-Marseille Univ, C2VN, INSERM, INRAE, Hôpital de la Conception, Service de Néphrologie et Transplantation rénale, Marseille, France; 6 Hôpital de Poitiers, Service de Néphrologie, Hémodialyse et Transplantation Rénale, Poitiers, France; 7 Hôpital de Purpan, Laboratoire d’Immunologie – Institut Fédératif de Biologie, Toulouse, France; 8 Hôpital de la Timone, Biogénopôle – Immunologie, Marseille, France; 9 AP-HP, Hôpital Tenon, Service de Néphrologie et Dialyse, Paris, France; 10 Sorbonne Université, INSERM CIMI U1135, AP-HP, Hôpital Pitié-Salpêtrière, Service de Néphrologie, Unité de Transplantation Rénale, Paris, France; 11 AP-HP, Hôpital Saint-Louis, Service de Néphrologie et Transplantation Rénale, Paris, France; 12 AP-HP, Hôpital Bichat, Service de Néphrologie, Paris, France; 13 AP-HP, Université Paris Cité, INSERM U1149, Centre de Recherche sur l'Inflammation, Paris, France; 14 Aix-Marseille Univ, C2VN, INSERM, INRAE, Hôpital de la Timone, Service d’Anatomie Pathologie, Marseille, France; 15 Université Paris Cité, INSERM U790, Centre de Recherche Cardiovasculaire de Paris, France; 16 AP-HP, Hôpitaux Universitaires Henri Mondor, Service de Néphrologie et Transplantation, Créteil, France; 17 Université Paris Est-Créteil, INSERM U955, Institut Mondor de recherche Biomédicale (IMRB), Créteil, France; 18 Hôpitaux de Rouen, Service de Néphrologie, Rouen, France; 19 CHU de Dijon, Service de Néphrologie, Dijon, France; 20 Univ. Lille, Inserm, CHU Lille, Département de Médecine Interne et Immunologie Clinique, Centre de référence des maladies autoimmunes systémiques rares du Nord, Nord-Ouest, Méditerranée et Guadeloupe (CeRAINOM), U1286 – INFINITE—Institute for Translational Research in Inflammation, Lille, France; 21 Hôpital Claude Huriez, Service de Néphrologie, Lille, France; 22 Hôpitaux Universitaires de Strasbourg, Service de Néphrologie, Dialyse et Transplantation, Strasbourg, France; 23 Centre Hospitalier Universitaire de Nantes, Service de Néphrologie et Immunologie, Nantes, France; 24 AP-HP, Hôpital de Bicêtre, Service d’Anatomie Pathologique, Le Kremlin-Bicêtre, France; 25 AP-HP, Hôpital Pitié-Salpêtrière, Service de Médecine Interne 2, Paris, France; 26 AP-HP, Hôpital Pitié-Salpêtrière, Service d’Immunologie, Paris, France; 27 AP-HP, Hôpital Cochin, Service de Médecine Interne, Paris, France

**Keywords:** cryofibrinogen, glomerular disease, monoclonal gammopathy of renal significance, membranoproliferative glomerulonephritis, nephrotic syndrome, thrombotic microangiopathy

## Abstract

**Introduction:**

Cryofibrinogen-associated nephropathy (CFN) is a very rare disease. Only few data are available about the clinicopathological presentation and treatment outcomes.

**Methods:**

Patients with cryofibrinogenemia (CF) diagnosed in French expert laboratories, and kidney biopsy findings suggestive of CFN (pauci-immune membranoproliferative glomerulonephritis [MPGN], thrombotic microangiopathy [TMA] and/or indirect signs of ischemia) were retrospectively included. Estimated glomerular filtration rate (eGFR), urinary protein-to-creatinine ratio (UPCR), and specific treatments were collected. Renal response (RR) was defined as a reduction of UPCR to < 0.5 g/g (or decrease > 50% if > 3 g/g at baseline) and an improvement of eGFR > 30% (if < 60 ml/min per 1.73 m^2^ and acute kidney injury (AKI) at baseline).

**Results:**

Among 2545 patients with CF, 232 (9%) underwent kidney biopsy, and only 28 (1%) had histological findings suggestive of CFN. Ten patients (36%) were female, and median age was 62 (interquartile range [IQR]: 49–71) years. eGFR at diagnosis was 21 (14–44) ml/min per 1.73 m^2^. Median UPCR was 3.30 (IQR: 1.52–4.85) g/g. AKI (78%) and nephrotic syndrome (41%) were frequent. Nine patients had an essential form, whereas 19 had a secondary form. MPGN was the most frequent pattern, with double contours (57%), nodular mesangial sclerosis (30%), mesangial (41%), endocapillary (56%) and extracapillary (11%) hypercellularity, and interstitial immune infiltration (41%). Thrombi were found in 43% of cases. Strikingly, 58% of the secondary forms were associated with a monoclonal gammopathy (MG). Patients with MG had more frequent skin manifestations (*P* = 0.002), endocapillary (*P* = 0.002), interstitial (*P* = 0.041) infiltration, and capillary thrombi (*P* = 0.012), and tended to have more frequent complement activation (*P* = 0.14). Sixty percent of patients were treated with various regimens of immunosuppressants (IS). After a mean follow-up of 476 (± 92) days, 65% of patients had an RR. Higher baseline eGFR (*P* = 0.04) and use of IS (*P* = 0.03) were predictive of RR. B-cell or plasma-cell depletion was effective in most cases associated with MG (80%).

**Conclusion:**

Our study, to the best of our knowledge, described for the first time the prevalence and the clinicopathological spectrum of CFN, which might be a very rare and underrecognized form of MG of renal significance presenting with pauci-immune MPGN ot TMA. IS are effective in most cases.

## Graphical abstract

**Figure:**
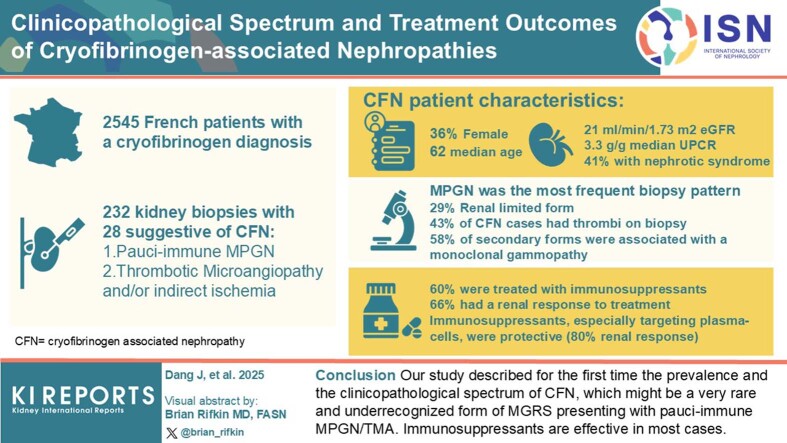

Cryofibrinogenemia is a rare and potentially life-threatening disease, affecting both females and males around the fifth decade of life, and was described for the first time by Korst and Kratochvil in 1955.^1^ CF is defined and caused by the cold-inducible precipitation of fibrinogen in the vessels; however, this cryoprecipitate also includes fibrin, and fibrin-degradation products, with or without Ig.^2^ Unlike cryoglobulin (CG), cryofibrinogen is able to precipitate only in cooled plasma at 4 °C but not in serum and also dissolves upon warming at 37 °C.^3^

Currently, CF can be classified into a primary (or essential) form; or a secondary form in association with various autoimmune disorders, solid organ and hematologic malignancies, MG of undetermined significance, and acute or chronic infections, such as hepatitis C virus.^4^, ^5^, ^6^ The clinical manifestations of CF are diverse, ranging from asymptomatic to thromboembolic phenomenon. The skin is the most common target organ (80% of cases), with purpura, livedo reticularis, and Raynaud’s phenomenon being the most frequent symptoms, often triggered by cold exposure (40% of cases). Ulceration, gangrene, and ischemic necrosis can occur in more severe cases. These lesions typically appear at the following distal extremities: buttocks, hands, feet, ears, and nose. Large arteries or veins thrombosis may occur in 20% to 40% of patients, including lower limbs, digestive tract, myocardium, and central nervous system.^4^^,^^5^^,^^7^ Although its pathophysiology remains unclear, there has been a renewed and growing interest since CF has also been suspected to cause chilblains in patients during the COVID-19 pandemic.^8^^,^^9^

The kidney is also a target organ for cryofibrinogen. According to Terrier *et al.*,^7^ CF was detected in up to 11% of patients admitted to a single-hospital for management of kidney disorders, with kidney arterial thrombosis in some cases. Saadoun *et al.*^4^ reported a kidney involvement, defined by the presence of proteinuria, hematuria or elevated serum creatinine, in 13% of patients with CF, most of whom had a secondary form (mainly associated with systemic lupus).^4^ More recently, attention has been paid to a peculiar and unrecognized form of CF-related glomerulonephritis: Sethi *et al.*,^10^ and others, described a few cases of MPGN, with double contours, mesangial and/or endocapillary hypercellularity, direct observation of eosinophilic plugs in small vessels in some cases, and no or weak segmental capillary wall fibrinogen staining, without significant deposition of Ig or complement.^11^ This histological pattern may be suggestive of chronic TMA, and the absence of immune complexes allows their distinction from CG glomerulonephritis or other rare diseases such as fibrillary or immunotactoid glomerulopathies.^10^ Electron microscopy can be helpful in difficult cases, with the identification of organized and distinctive intracapillary and/or subendothelial deposits of large tubular structures (around 150 nm of diameter) with prominent central bore and double or triple lamellations.^10^

The prevalence, characteristics and management of these different forms of CFN are unclear. This retrospective multicenter study was designed to determine the following: (i) the clinical and pathological spectrum of CFN and (ii) their outcomes.

## Methods

### Study Population and Data Collection

All adult patients with CF diagnosed in French expert laboratories, from January 2005 to December 2023, were considered for inclusion in this retrospective observational study. Patients with kidney biopsy were identified through the electronic database from the pathology departments. The diagnosis of CFN was defined by the following: (i) the presence of cryofibrinogen, together with the identification of (ii) a pauci-immune MPGN, TMA and/or indirect signs of ischemia (folding of the glomerular capillary walls, collapse of the capillary tuft) suggestive of kidney vessels thrombosis, on kidney biopsy, (iii) in the absence of other differential diagnosis. Demographic, clinical, biological and histological findings, and treatment modalities were collected from medical charts. Patients with missing data regarding renal parameters (eGFR and/or UPCR) were excluded. Baseline eGFR was defined from the last serum creatinine value obtained within the 3 months preceding the diagnosis of CFN. Histological findings have been reviewed by a single pathologist. This study was performed in accordance with the Declaration of Helsinki, and was approved by our local ethics committee (Créteil) and by the French Comité National de l’Informatique et des Libertés (CNIL number 2236784v0)

### Cryoproteins and Kidney Function Assessment

Analysis of cryoproteins was performed as previously described.^4^ eGFR was calculated based on serum creatinine using the Chronic Kidney Disease – Epidemiology Collaboration formula.^12^ Significant proteinuria was defined by a UPCR ≥ 0.5 g/g on a spot urine sample. eGFR and UPCR were collected at the time of CFN diagnosis and during follow-up.

### RR to Therapy

RR was defined as a reduction of UPCR to < 0.5 g/g, or a decrease of > 50% if baseline UPCR was > 3 g/g. For patients with an eGFR < 60 ml/min per 1.73 m^2^ and an AKI at diagnosis, an improvement of eGFR ≥ 30% was also needed to fulfill the criteria of RR.^13^ AKI was defined according to the Kidney Disease: Improving Global Outcomes guidelines.^14^

### Statistical Analysis

Continuous variables were described using mean values with SD or median values with IQR (IQR is 25–75 percentile) as appropriate, whereas categorical variables were presented as counts and percentages. Frequency differences for qualitative variables were compared in chi square tests or Fisher exact tests, as appropriate. Nonparametric tests were used for quantitative variables. All tests were 2-tailed and *P*-value of < 0.05 was considered as statistically significant. Survival was estimated using the Kaplan–Meier method and compared with the log-rank test. Statistical analyses were performed using GraphPad Prism 8.0 (La Jolia, CA).

## Results

### Demographic and Clinical Characteristics

**Figure 1:**
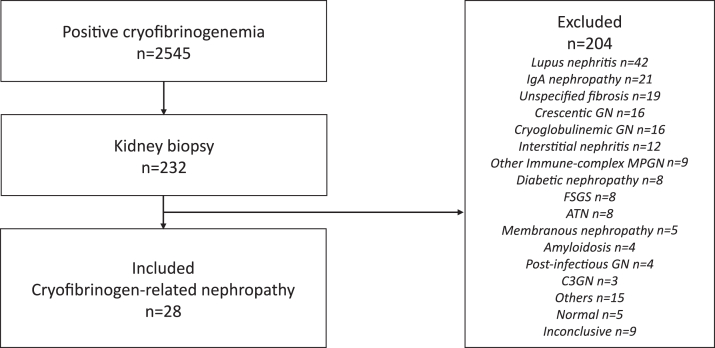
Flow-chart. Among the 2545 cases of cryofibrinogenemia diagnosed at French expert laboratories between 2005 and 2023, 232 patients had a kidney biopsy performed. After the exclusion of 204 patients with differential diagnoses, 28 patients with suspected cryofibrinogen-related nephropathies were included in this study. ATN, acute tubular necrosis; C3GN, C3 glomerulopathy; FSGS, focal segmental glomerulosclerosis; GN, glomerulonephritis; MPGN, membranoproliferative glomerulonephritis.

From January 2005 to December 2023, 2545 patients had a detectable CF diagnosed at French expert laboratories. Among them, 232 had a kidney biopsy. After exclusion of patients with differential diagnosis of nephropathies, 28 cases of CFN (1%) were finally included (Figure 1). Baseline characteristics are summarized in Table 1. Ten patients (36%) were female. The median age was 62 (IQR:49–71) years. Past medical history included diabetes mellitus in 3 of 28 cases (11%), malignancy in 7 of 28 cases (25%), autoimmune disease in 2 of 28 cases (7%), and chronic infection (HIV, *n* = 3; hepatitis C virus, *n* = 4; hepatitis B virus, *n* = 2; 3 patients had coinfections; all patients, except for 1 patient with chronic hepatitis C, were treated and infections were controlled at baseline) in 6 of 28 (21%) cases. CF was considered essential in 9 of 28 cases (32%), and secondary in 19 of 28 cases (68%). Suspected underlying causes of CF were an MG in 11 of 19 cases (58%), a progressive solid cancer (breast, *n* = 1; ovary, *n* = 1; prostate, *n* = 1; and lung, *n* = 2), an acute infection (recurrent dermohypodermitis, *n* = 1; prosthetic joint infection by *Staphylococcus aureus* and *Staphylococcus lugdunensis*, *n* = 1) or a chronic infection (hepatitis C virus) in 5 of 19 (26%), 2 of 19 (11%), and 1 of 19 (5%) cases, respectively. Systemic manifestations included fever (3/28, 11%), skin manifestations (16/28, 57%), arthralgia (5/28, 18%), peripheral neuropathy (5/28, 18%), acute coronary syndrome (2/28, 7%), stroke (3/28, 11%), gastrointestinal ischemia (2/28, 7%), and venous thrombosis (2/28, 7%). Eight patients (29%) had a kidney-limited form of CF.

**Table 1:** Clinical characteristics of patients with CFN at the time of diagnosis

Variables	All patients *N* = 28 (100%)	Essential form *n* = 9 (32%)	Monoclonal gammopathy^a^*n* = 11 (39%)	Other secondary forms^a^*n* = 8 (29%)	*P*-value
Demographic characteristics	Median (IQR) or *n* (%)	Median (IQR) or *n* (%)	Median (IQR) or *n* (%)	Median (IQR) or *n* (%)	
Age (yrs)	62 (49–71)	49 (47–64)	68 (42–78)	63 (52–71)	0.38
Female	10/28 (36)	3/9 (33)	3/11 (27)	4/8 (50)	0.58
BMI (kg/m^2^)	24.2 (21.2–27.3)	24.5 (21.2–27.3)	22.9 (19.6–28.7)	24.1 (22.6–27.5)	0.90
Systemic manifestations	Median (IQR) or *n* (%)	Median (IQR) or *n* (%)	Median (IQR) or *n* (%)	Median (IQR) or *n* (%)	
Kidney-limited form	8/28 (29)	4/9 (44)	1/10 (10)	3/8 (38)	0.18
Fever > 38.5 °C	3/28 (11)	0/9 (0)	2/11 (18)	1/8 (13)	0.42
Arthralgia	5/28 (18)	0/9 (0)	3/11 (27)	2/8 (25)	0.24
Peripheral neuropathy	5/28 (18)	1/9 (11)	3/11 (27)	1/8 (13)	0.58
Purpura or livedo	14/28 (50)	2/9 (22)	10/11 (91)	2/8 (25)	0.002
Raynaud phenomenon	4/28 (14)	1/9 (11)	3/11 (27)	0/8 (0)	0.23
Skin ulceration / necrosis	4/28 (14)	0/9 (0)	1/11 (9)	3/8 (38)	0.07
Acute coronary syndrome	2/28 (7)	2/9 (22)	0/11 (0)	0/8 (0)	0.10
Stroke	3/28 (11)	2/9 (22)	0/11 (0)	1/8 (13)	0.27
Gastrointestinal ischemia	2/28 (7)	1/9 (11)	0/11 (0)	1/8 (13)	0.50
Venous thrombosis	2/28 (7)	1/9 (11)	1/11 (9)	0/8 (0)	0.64
Biological parameters	Median (IQR) or *n* (%)	Median (IQR) or *n* (%)	Median (IQR) or *n* (%)	Median (IQR) or *n* (%)	
Fibrinogen level (g/l)	5.6 (4.7–6.9)	5.2 (4.5–6.5)	5 (2.2–6.9)	6.7 (5.8–7.5)	0.13
Biological signs of TMA	0/16 (0)	0/7 (0)	0/5 (0)	0/4 (0)	
Complement activity					
Normal	21/26 (81)	8/9 (89)	7/11 (64)	6/6 (100)	0.14
Low C3	1/26 (4)	0/9 (0)	1/11 (9)	0/6 (0)	
Low C4	2/26 (8)	0/9 (0)	2/11 (18)	0/6 (0)	
Low C3 and C4	2/26 (8)	1/9 (11)	1/11 (9)	0/6 (0)	
Associated cryoglobulin	10/28 (36)	5/9 (56)	2/11 (18)	3/8 (38)	0.22
Polyclonal	6/28 (21)	2/5	2/2	2/3	
Monoclonal^b^	4/25 (16)	3/5	0/2	1/3	

BMI, body mass index; CFN, cryofibrinogen-related nephropathy; IQR, interquartile range; TMA, thrombotic microangiopathy.

Data are presented as *n* (%) of patients or median (IQR). Analyses were performed with chi-square tests, Fisher exact tests, or *t* tests or Mann-Whitney tests, as appropriate. Values of *P* < 0.05 were considered statistically significant, all tests were 2-tailed.

a Suspected underlying cause of cryofibrinogenemia were a monoclonal gammopathy in 11 of 19 cases (58%), or a progressive solid cancer (breast, *n* = 1; ovary, *n* = 1; prostate, *n* = 1; and lung, *n* = 2), an acute infection (recurrent dermohypodermitis, *n* = 1; and prosthetic joint infection by *Staphylococcus aureus* and *Staphylococcus lugdunensis*, *n* = 1) or a chronic infection (hepatitis C virus) in 5 of 19 (26%), 2 of 19 (11%) and 1 of 19 (5%) cases, respectively.

b IgM kappa (*n* = 3), IgG kappa (*n* = 1).

### Laboratory Features and Histopathology

**Figure 2:**
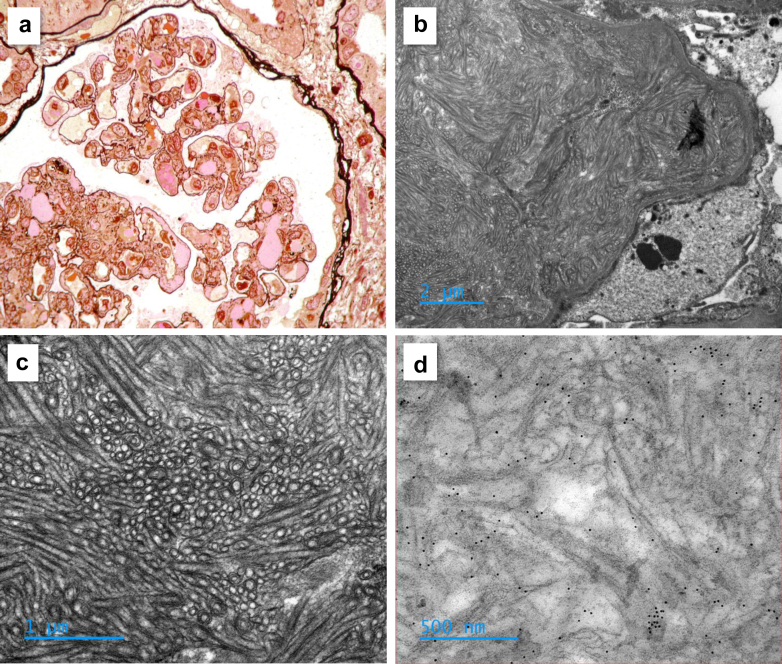
Cryofibrinogen-associated glomerulonephritis (CFG). (a) Kidney biopsy of a 69-year-old male patient with a CFG, presenting with nephrotic syndrome, mild acute kidney injury and a monoclonal IgG Kappa. Light microscopy analysis revealed focal mesangial sclerosis, endocapillary mononuclear hypercellularity, double contours and endoluminal thrombi. (b and c) Electron-microscopy analysis of a kidney biopsy of a 71-year-old male patient, diagnosed with CFG associated with a monoclonal IgG Kappa, revealing subendothelial deposition of organized and large tubular structures (around 150 nm of diameter) with prominent central bore and double or triple lamellations, suggestive of CFG. (d) Immuno-electron microscopy analysis using anti-kappa, anti-lambda and anti-fibrinogen antibodies was exclusively positive for fibrinogen in this latter patient.

Renal parameters are detailed in Table 2 and Supplementary Table S1. Median baseline eGFR was 53 (38–90) ml/min per 1.73 m^2^ and was < 60 ml/min per 1.73 m^2^ in 13 of 23 patients (57%). Patients presented with AKI in 18 of 23 cases (78%), and 2 of them needed renal replacement therapy at diagnosis. Median UPCR was 3.30 (IQR: 1.52–4.85) g/g, > 0.5 g/g in 26 of 28 patients (93%), and nephrotic syndrome was found in 11 of 27 cases (41%). Microscopic hematuria was documented in 23 of 28 cases (82%). Histopathological characteristics are detailed in Table 2 and Supplementary Table S2. For 1 case, the biopsy could not be retrieved because the record was too old (2005), and the interpretation was based on the pathologist’s report from that time. MPGN was the predominant pattern. Capillary wall remodeling with double contours was observed in 57% of cases, nodular mesangial sclerosis in 30% of cases; and mesangial, endocapillary or extracapillary hypercellularity in 41%, 56%, and 11% of cases, respectively. Intraluminal thrombi were found in 43% of cases. Capillary tuft retraction, suggestive of kidney vessels thrombosis, was seen in 56% of cases. No significant immune deposits (Ig, complement) were found. Ten patients had concomitant CG in serum, without Ig deposits. Electron microscopy analysis was performed in 8 cases. Granular nonorganized subendothelial dense deposits were observed in 4 cases, 1 was noncontributive, and 1 showed a TMA. In 2 cases, typical organized mesangial and subendothelial deposits of large tubular structures were found, suggestive of CFN, and fibrinogen was exclusively identified by immunogold staining in 1 case (Figure 2). Of note, none of the patients showed biological features of TMA (mechanic hemolysis).

**Table 2:** Renal parameters and histopathological characteristics of patients with CFN at the time of diagnosis

Variables	All patients *N* = 28 (100%)	Essential form *n* = 9 (32%)	Monoclonal gammopathy^a^*n* = 11 (39%)	Other secondary forms^a^*n* = 8 (29%)	*P*-value
Clinical and biological parameters	Median (IQR) or n (%)	Median (IQR) or *n* (%)	Median (IQR) or *n* (%)	Median (IQR) or *n* (%)	
High blood pressure > 140/90 mm Hg	21/28 (75)	8/9 (89)	8/11 (73)	5/8 (63)	0.44
Lower limbs edema	10/28 (36)	3/9 (33)	3/11 (27)	4/8 (50)	0.58
Macroscopic hematuria	5/28 (18)	1/9 (11)	2/11 (18)	2/8 (25)	0.76
Microscopic hematuria	23/28 (82)	7/9 (78)	11/11 (100)	5/8 (63)	0.09
UPCR (g/g)	3.30 (1.52–4.85)	3 (1.36–4.83)	3.60 (1.50–3.86)	3.41 (1.16–4.98)	0.99
Significant proteinuria^b^	26/28 (93)	8/9 (89)	11/11 (100)	7/8 (88)	0.49
Nephrotic syndrome	11/27 (41)	3/9 (33)	5/10 (50)	3/8 (38)	0.74
eGFR at baseline (ml/min per 1.73 m^2^)	53 (38–90)	46 (38–57)	84 (48–114)	41 (24–90)	0.13
eGFR < 60 ml/min per 1.73 m^2^	13/23 (57)	5/6 (83)	3/10 (30)	5/7 (71)	0.07
eGFR at diagnosis (ml/min per 1.73 m^2^)	21 (14–44)	19 (15–24)	45 (18–76)	14 (8–31)	0.046
AKI at diagnosis	18/23 (78)	6/6 (100)	7/10 (70)	5/7 (71)	0.32
AKI stage 1	4/18 (22)	1/6 (17)	2/7 (29)	1/5 (20)	0.87
AKI stage 2	9/18 (50)	4/6 (67)	4/7 (57)	1/5 (20)	0.27
AKI stage 3	5/18 (28)	1/6 (17)	1/7 (14)	3/5 (60)	0.17
Dialysis	2/28 (7)	0/9 (0)	1/11 (9)	1/8 (13)	0.58
Histopathological characteristics	Median (IQR) or *n* (%)	Median (IQR) or *n* (%)	Median (IQR) or *n* (%)	Median (IQR) or *n* (%)	
Glomerular lesions					
Double contours	16/28 (57)	4/9 (44)	10/11 (91)	2/8 (25)	0.011
Mesangial thickening	14/27 (52)	2/9 (22)	7/10 (70)	5/8 (63)	0.09
Nodular mesangial sclerosis	8/27 (30)	1/9 (11)	6/10 (60)	1/8 (13)	0.030
Mesangial hypercellularity	11/27 (41)	4/9 (44)	5/10 (50)	2/8 (25)	0.54
Endocapillary hypercellularity	15/27 (56)	3/9 (33)	10/10 (100)	2/8 (25)	0.002
Extracapillary hypercellularity	3/27 (11)	1/9 (11)	2/10 (20)	0/8 (0)	0.41
FSGS	9/27 (33)	4/9 (44)	5/10 (50)	0/8 (0)	0.056
Capillary tuft retraction	15/27 (56)	4/9 (44)	4/10 (40)	7/8 (88)	0.09
Thrombi	12/28 (43)	5/9 (56)	6/11 (55)	1/8 (13)	0.12
Arteriolar thrombi	6/27 (22)	5/9 (56)	1/10 (10)	0/8 (0)	0.012
Glomerular capillary thrombi	7/28 (25)	0/9 (0)	6/11 (55)	1/8 (13)	0.012
Interstitial fibrosis >25%	13/27 (48)	5/9 (56)	3/10 (30)	5/8 (63)	0.34
Interstitial immune infiltration	11/27 (41)	3/9 (33)	7/10 (70)	1/8 (13)	0.041

AKI, acute kidney injury; CFN, cryofibrinogen-related nephropathy; CKD, chronic kidney disease; eGFR, estimated glomerular filtration rate usingthe CKD-EPI (Chronic Kidney Disease EPIdemiology collaboration) formula; FSGS, focal segmental glomerulosclerosis; UPCR, urinary protein-to creatinine-ratio.

Data are presented as *n* (%) of patients or median (IQR). Analyses were performed using chi-square tests, Fisher exact tests, or *t* tests or Mann-Whitney tests, as appropriate. Values of *P* < 0.05 were considered statistically significant, all tests were 2-tailed.

a Suspected underlying cause of cryofibrinogenemia were a monoclonal gammopathy in 11 of 19 (58%) cases, or a progressive solid cancer (breast, *n* = 1; ovary, *n* = 1; prostate, *n* = 1; and lung, *n* = 2), an acute infection (recurrent dermohypodermitis, *n* = 1; and prosthetic joint infection by *Staphylococcus aureus* and *Staphylococcus lugdunensis*, *n* = 1) or a chronic infection (hepatitis C virus) in 5 of 19 (26%), 2 of 19 (11%) and 1 of 19 (5%) cases, respectively.

b Urine protein-to-creatinine ratio > 0.5 g/g.

### Treatments and Outcomes

**Figure 3:**
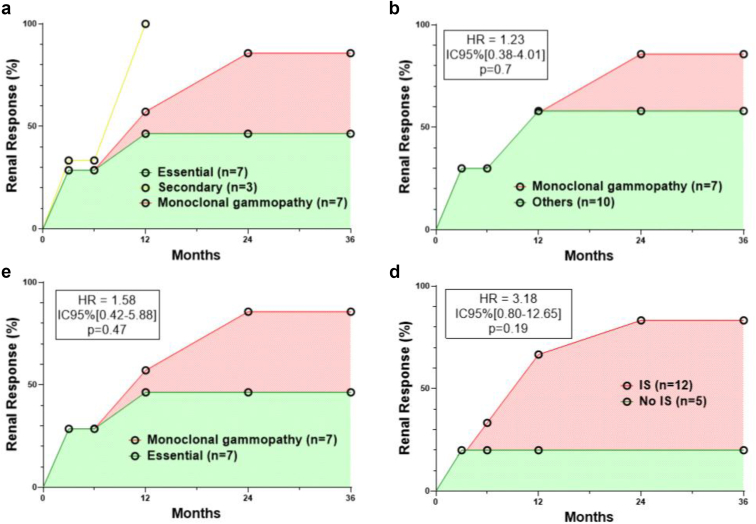
Renal response. Cumulative renal response according to the form of cryofibrinogenemia and the treatment or not by immunosuppressive agents, defined by urine protein-to-creatinine ratio < 0.5 g/g, or a decrease of > 50% if baseline urine protein-to-creatinine ratio was > 3 g/g, together with an improvement of estimated glomerular filtration rate of 30% if estimated glomerular filtration rate was < 60 ml/min per 1.73 m^2^ with acute kidney injury at diagnosis. HR, hazard ratio; IC, confidence interval; IS, immunosuppressants.

Treatment modalities data at diagnosis were available for 25 patients and consisted of IS in 15 (60%) of them. After a mean follow-up of 476 (± 92) days, 5 patients died (2 in the essential form group, 1 in the MG group, and 2 in the other secondary form group). Among the 17 patients with renal parameters follow-up, 11 of 17 (65%) had a RR (3/7 in the essential forms, 6/7 in the patients with MG, and 2/3 in the other secondary forms). Analyses of factors associated with RR are detailed in Table 3 and Figure 3. Systemic manifestations, complement activation or the presence of CG were not different in patients with or without RR. The presence of endocapillary hypercellularity (*P* = 0.10) and a higher baseline eGFR (*P* = 0.04) were more predictive of RR. Use of IS was protective (*P* = 0.03). IS modalities were heterogeneous (detailed in Supplementary Table S3) and consisted of corticosteroids (CS), cyclophosphamide (CYC), calcineurin inhibitor, rituximab, bortezomib, daratumumab, isatuximab, lenalidomide or also eculizumab and plasma exchange in a few cases. Four of the 5 patients (80%) treated with IS (2 with CS only, 1 with CS and CYC, 1 with CS and eculizumab) in the essential and other secondary form groups, had an RR within 3 to 12 months. The remaining 1 had no response after 2 years under eculizumab alone. Only 1 of the 5 patients (20%) treated with nephroprotective measures alone had a RR.

**Table 3:** Factors associated with renal response in patients with CFN

Variables	Renal Response *n* = 11 (65%)	No Renal Response *n* = 6 (35%)	*P*-value
Clinical and biological characteristics			
Age (yrs)	51 (46–68)	58 (44–74)	0.61
Female	4/11 (36)	1/6 (17)	0.60
Kidney-limited form	2/11 (18)	3/6 (50)	0.28
Complement activation	2/11 (18)	2/6 (33)	0.58
Nephrotic syndrome	6/11 (55)	1/5 (20)	0.31
eGFR at baseline (ml/min per 1.73 m^2^)	81 (54–109)	40 (31–60)	0.04
eGFR at diagnosis (ml/min per 1.73 m^2^)	19 (10–49)	24 (21–34)	0.61
Associated cryoglobulin	5/11 (46)	4/6 (67)	0.62
Essential form	3/11 (27)	4/6 (67)	0.16
Monoclonal gammopathy	6/11 (55)	1/6 (17)	0.30
Histopathological characteristics			
Double contours	8/11 (73)	3/6 (50)	0.60
Mesangial thickening	5/11 (46)	2/5 (40)	0.99
Nodular mesangial sclerosis	3/11 (27)	1/5 (20)	0.99
Mesangial hypercellularity	7/11 (64)	2/5 (40)	0.60
Endocapillary hypercellularity	9/11 (82)	1/5 (20)	0.10
Extracapillary hypercellularity	2/11 (18)	0/5 (0)	0.99
FSGS	5/11 (46)	1/5 (20)	0.59
Capillary tuft retraction	6/11 (55)	3/5 (60)	0.99
Thrombi	6/11 (55)	2/6 (33)	0.62
Interstitial fibrosis > 25%	4/11 (36)	3/5 (60)	0.60
Interstitial immune infiltration	5/11 (46)	2/5 (40)	0.99
Treatments			
Immunosuppressants	10/11 (91)	2/6 (33)	0.03

CFN, cryofibrinogen-related nephropathy; eGFR, estimated glomerular filtration rate by CKD-EPI (Chronic Kidney Disease EPIdemiology collaboration) formula; FSGS, focal segmental glomerulosclerosis.

Data are presented as *n* (percentage) of patients or median (IQR). Analyses were performed with chi-square tests, Fisherexact tests, or *t* tests or Mann-Whitney tests, as appropriate. Values of *P* < 0.05 were considered statistically significant, all tests were 2-tailed.

### Focus on Patients With CFN and MG

**Figure 4:**
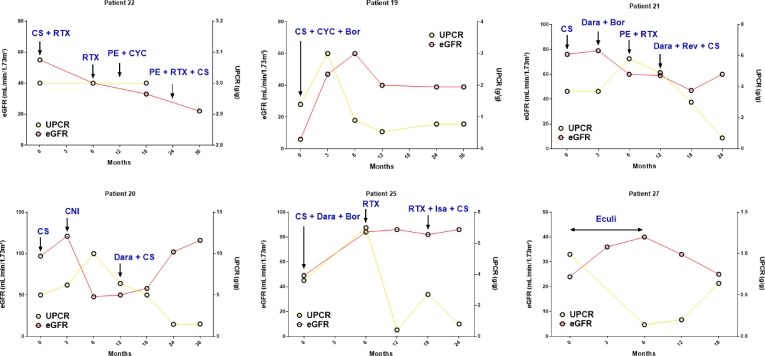
Treatment outcomes of patients with cryofibrinogen-associated nephropathy and monoclonal gammopathy. Evolution of eGFR and UPCR under treatments of patients with cryofibrinogen-associated nephropathy and monoclonal gammopathy. Bor, bortezomib; CNI, calcineurin inhibitor; CS, corticosteroids; CYC, cyclophosphamide; Dara, daratumumab; Eculi, eculizumab; eGFR, estimated glomerular filtration rate; Isa, isatuximab; PE, plasma exchange; Rev, Revlimid; RTX, rituximab; UPCR, urine protein-to-creatinine ratio.

Because of the unexpectedly high prevalence of MG in this cohort (39% in total, and 58% of the secondary forms), we analyzed patients with MG separately. These patients tended to be older (median age of 68 vs. 49 years in the essential form group and 63 years in the other secondary form group, respectively, *P* = 0.38); had more frequently systemic manifestations, especially purpura and livedo (91%, 22%, and 25%, respectively, *P* = 0.002), biological complement activation (36%, 11%, and 0%, respectively, *P* = 0.14), and fewer associated CG (18%, 56%, and 38%, respectively, *P* = 0.22) (Table 1). Baseline renal function was better in this group (median eGFR of 84, 46, and 41 ml/min per 1.73 m^2^, respectively, *P* = 0.13). Microscopic hematuria (100%, 78% and 63%, respectively, *P* = 0.09) and renal lesions such as double contours (91%, 44%, and 25%, respectively, *P* = 0.011), nodular mesangial sclerosis (60%, 11%, and 13%, respectively, *P* = 0.03), endocapillary hypercellularity (100%, 33%, and 25%, respectively, *P* = 0.002) and interstitial immune infiltration (70%, 33%, and 13%, respectively, *P* = 0.041) were more frequent. Patients with the essential form (56%) or MG (55%) had endoluminal thrombi more frequently than those with other secondary form (13%); however, patients with the essential form had more frequently arteriolar thrombi, whereas those with MG had more frequently glomerular capillary thrombi (*P* = 0.012) (Table 2). MG was an IgGƙ, an IgGλ, and an IgAƙ in 7 of 11 (64%), 3 of 11 (27%) and 1 of 11 (9%) of cases, respectively. Myelogram was available for 6 patients, revealing 10% dystrophic plasmacytosis in 2 of them, without histological evidence for cast nephropathy on kidney biopsy (Supplementary Tables S1 and S2). RR tended to be more frequent in patients with MG than in those with the essential form (hazard ratio = 1.58, 95% confidence interval: [0.42–5.88], *P* = 0.47), the effect seeming mostly driven by the use of IS agents (hazard ratio = 3.18, 95% confidence interval: [0.80–12.65], *P* = 0.19) (Figure 3). Details of renal outcomes and treatment modalities are described for the 6 patients with MG treated with IS agents and adequate follow-up in Figure 4, Supplementary Table S3, and Supplementary Data.

## Discussion

To our knowledge, this is the largest retrospective series of CFN diagnosed in expert French centers. It delineates the prevalence, the clinical and pathological spectrum of CFN and describes renal outcomes under treatments. We also report an unexpectedly high prevalence of MG, which is associated with distinct features and outcomes.

### Prevalence

As expected, CFN is rare: only 9% of patients with CF underwent kidney biopsy, and merely 1% had lesions consistent with CFN. The prevalence of CF itself remains unclear; however, up to 3% to 9% of healthy individuals may have small amounts of detectable cryofibrinogen, rarely exceeding 0.05 g/l, whereas an overall prevalence of 3% to 50% has been reported in hospitalized patients especially those with symptoms suggestive of cryopathy.^5^^,^^15^, ^16^, ^17^, ^18^ Furthermore, diagnostic accuracy is challenged by sample handling issues such as improper temperature control or delayed processing. Altogether, it is difficult to make a right and prompt diagnosis of CFN. However, the fact that one-third of our patients exhibited kidney-limited disease highlights the need for nephrologist awareness.

### Clinical and Pathological Spectrum

Knowledge about CF among nephrologists has been limited to very few cases of thrombotic phenomenon leading to renal vessels thrombosis or calciphylaxis.^7^^,^^19^ In our cohort, signs of renal ischemia (e.g., capillary walls folding, tuft retraction) were observed in 56% of cases, although perfusion imaging was rarely performed. CFN more commonly presents as severe nephritic syndrome with AKI (78%) and nephrotic range proteinuria (41%), rather than overt vascular thrombosis. In fact, pauci-immune MPGN and TMA were the most common histological patterns. The existence of this cryofibrinogen-associated glomerular disease has been suggested for the first time in 2007 by Singh *et al.*,^20^ who reported the case of a patient with nephrotic syndrome and a skin-limited CF diagnosed 5 years earlier. Kidney biopsy showed a typical “chronic TMA-like” pauci-immune MPGN with very rare electron dense subendothelial deposits. In 2016, Sethi *et al.*^10^ finally provided for the first time, a detailed characterization of 2 cases of cryofibrinogen-associated MPGN. In both cases, kidney biopsy showed a similar pattern of MPGN with rare scattered intracapillary eosinophilic deposits, endocapillary hypercellularity, and only weak segmental capillary wall fibrinogen and C3 staining. Immunofluorescence studies, even following pronase digestion, failed to reveal masked Ig deposits. Ultrastructural studies were distinctively unique and characterized by organized subendothelial deposits of large-bore with multilayered tubular structures, admixed with curvilinear deposits set in a matrix. These fluorescent and ultrastructural patterns allow their distinction from CG glomerulonephritis or other rare diseases such as fibrillary, immunotactoid or fibronectin glomerulopathies.^10^ Ultrastructural studies of the cryoprecipitate extracted from the plasma showed identical characteristics of those seen on the kidneys. Few other case reports have been published later on, further highlighting the usefulness of laser microdissection and mass spectrometry in difficult cases because of an inconstant aspect of deposits at electron microscopy.^11^^,^^21^^,^^22^ Likewise, typical ultrastructural characteristics described by Sethi were found in only 2 of 8 biopsies studied in our cohort.

### Treatment Outcomes

Only few data are available for the therapeutic management of CF. In addition to cold exposure avoidance, patients can be treated with aspirin, anticoagulants, and streptokinase or anabolic steroids for thrombotic forms, but also IS in secondary or severe forms, including CS, CNI or CYC, and PE, all with various degrees of success and evidence.^23^, ^24^, ^25^ Complete remission is expected in three-quarters of patient, with 40% experiencing relapses.^4^ As for the 13 published cases of CFN, renal outcomes were either not reported, or poor in most cases, except for 2 patients treated with high dose CS or combination of CS and CYC.^10^^,^^11^^,^^20^, ^21^, ^22^^,^^26^, ^27^, ^28^ In our series, RR was achieved in 65% of cases. The most predictive factor was the use of IS. IS were rapidly effective in all but 1 case of essential and other secondary forms of CFN without MG, and were based on CS, usually at 1 mg/kg/d after pulse doses, in combination with CYC in 2 cases. The only refractory case was 1 patient treated with eculizumab alone. Supportive care alone was mostly ineffective.

### Association With MG

One striking observation of our study is the unexpectedly high prevalence of MG (39% overall, 58% among secondary CF cases). Among patients with CF from the Paris region, with or without kidney biopsy, 23% had MG. In comparison, the prevalence of MG is about 5% in the general population over 70 years. A possible link between CF and MG has been suggested in the 1970s ahead of clinical association.^29^ Zeuner *et al.*^30^ isolated an IgGƙ with antifibrinogen activity from the cryoprecipitate of a patient, undetectable by conventional serum or plasma electrophoresis, and without evidence for malignancy. Although CS and CYC were not successful in this patient, immunoadsorption had improved the skin and joint symptoms. Overall, among the 13 published cases of CFN, 8 were associated with a MG of undetermined significance (4 IgGƙ and 4 IgGλ). For the first time in 2023 (during the preparation of this manuscript), Gant *et al.*^31^ elegantly proved the role of MG in the cryoprecipitation of fibrinogen. They presented the case of a 39-year-old woman, with relapsing nephrotic syndrome due to a MPGN, and a cryoprecipitate including fibrinogen and an IgGλ, which was also minimally detected in serum, without evidence for malignancy at bone marrow investigation. Conventional IS therapy only resulted in temporary renal remission; therefore, subsequent plasma-cell targeting treatments were started, consisting of bortezomib/dexamethasone and high dose melphalan followed by autologous stem-cell transplantation, and resulted in a very good partial hematological and again, a temporary renal remission. The authors then performed complementary analysis to elucidate the role of the M-protein in her disease course. They found that mixing patient serum with donor plasma also resulted in the same cryoactivity, composed of both the M-protein and fibrinogen. Most importantly, patient plasma deprived of IgGλ could not induce this cryoactivity. Furthermore, eluted IgGλ from the patient could again form a precipitate at 4 °C when added to the plasma of a healthy control, suggesting that the cryoprecipitation was dependent of the M-protein. A subsequent relapse was successfully treated with lenalidomide. Altogether, the authors suggest that CFN might be defined as a new form of MG of renal significance. These findings have been subsequently confirmed by other authors.^32^^,^^33^ Interestingly, even in well-characterized cases with negative immunofluorescence studies, mass spectrometry analyses frequently reveal substantial Ig deposition, suggesting that CFN may be an immune complex–mediated disease after all.^10^^,^^11^^,^^33^ The question of why Ig are not detected in immunofluorescence studies remains unresolved. One possible explanation is that the coprecipitation of CF and Ig could mask epitopes that are usually recognized by antibodies in tissue.^33^ Conversely, our systematic exclusion of MPGN cases with Ig deposition may have led to the omission of true MG-related CFN cases. Furthermore, the presence of an MG with antifibrinogen affinity might be part of the missing link between CF and the immune cascade signalization. Because of its supposed inability to activate the immune system, cryofibrinogen was thought to induce very little inflammation or complement activation as compared to CG. Nevertheless, immune infiltration was frequent in our cohort, and biological complement consumption was observed. More particularly, patients with MG in our cohort had more frequently endocapillary hypercellularity and interstitial immune infiltration. Moreover, they tend to have more frequent biological consumption of complement as compared with other forms of CF. In contrast, CF might be a mechanism of the newly described prothrombotic nature of MG of undetermined significance, or the so-called monoclonal gammopathies of thrombotic significance.^34^ Finally, cryofibrinogen might also explain the few cases of TMA with concomitant MG that are not always associated with a significant activation of the alternate complement pathway.^35^ Interestingly, and accordingly to the hypothesis of MG of renal significance, IS were effective in our study in most patients with MG, especially when using plasma-cell depletion therapies, showing higher efficacy in refractory cases. Most importantly, Belizna *et al.*^36^ observed that 47% of the patients who were originally classified as having essential CF developed T-cell and B-cell lymphoma over 24 months of follow-up. Systemic manifestations were more frequent in these patients, as observed in our cohort among patients with MG. It is therefore probable that these patients could evolve toward overt hematological malignancies in the absence of adequate treatment. Long-term monitoring of these patients is thus necessary.

### Limitations

Our study has several limitations other than its retrospective nature. First, we exclusively included patients with histological features known to be caused by cryofibrinogen and may have excluded other unsuspected atypical cases. For example, 1 study by Nagy *et al.* reported a 74% prevalence of CF in a cohort of patients with biopsy-proven IgA nephropathy, but a causal effect had never been investigated.^37^ Interestingly, IgA nephropathy was particularly prevalent in our cohort (21/232 kidney biopsies). Second, levels of CF were not prospectively controlled in most of the patients. In a large French study, transient and mildly positive CF can occur in up to 16% of cases, mostly because of inflammatory syndrome, and is likely less pathogenic.^3^ Moreover, we cannot assess the prognostic value of CF levels in kidney function decline. Indeed, Nagy *et al.*^37^ found that patients with IgA nephropathy with persistent CF were more likely to have hematuria and to experience progressive deterioration of renal function. The various quantification methods used in the different laboratories prevent us from analyzing the correlation between the initial levels of circulating cryofibrinogen and the severity of kidney disease, although such a correlation has not been observed for extrarenal manifestations.^5^ Third, because of frequent TMA and complement consumption, thorough investigations of the alternate complement pathway are needed to help clarify mechanisms and guide therapeutic strategies. Of note, recent reports have shown that kidney-limited TMA can be observed quite frequently in complement-dependent atypical hemolytic and uremic syndrome.^38^^,^^39^ Fourth, although CG was expected to be frequently associated with cryofibrinogen (up to one-third of cases, as reported in Sarda-Kolopp and Miossec^3^) and no immune deposits have been found in our patients, we cannot exclude masked deposits at least in some cases. To definitely exclude cryoGN, repeated and simultaneous tests for cryofibrinogen and CG, pronase digestion on paraffin sections, and electron microscopy should be more systematically requested. The lack of a centralized review is a significant methodological limitation that should be emphasized. Fifth, one-third of the patients have been lost to follow-up, especially those with secondary forms associated with cancer. Sixth, renal perfusion imaging should be more frequently performed to exclude arterial or venous thrombosis and to help in discussing the necessity of an anticoagulation therapy. Finally, although immunosuppressive agents appear to be protective, treatment strategies were highly heterogeneous, making it difficult to draw clear recommendations.

### Conclusion and Future Directions

CFN is a likely underrecognized and potentially life-threatening condition. Our study points out the importance of an accurate and timely diagnosis to prevent kidney failure, which could be the only clinical presentation in one-third of the patients. We found that the prevalence of MG is unexpectedly high and that IS, including B-cell and especially plasma-cell depletion therapies, are particularly effective in these cases. These findings support the new hypothesis that CFN could be a rare form of MG of renal significance.

## Disclosure

All the authors declared no competing interests.

## Data Availability Statement

Data that support the findings are included within the manuscript.

## Author Contributions

Research idea was by JD. Diagnosis and follow-up of patients were by all the authors. Data acquisition was by JD. Data analysis and interpretation were by JD, BT, and MZ.

## Supplementary Material

Supplementary File (PDF) Supplementary Data. **Table S1.** Detailed renal clinical and biological characteristics of patients with CFN at the time of diagnosis. **Table S2.** Detailed renal histopathological characteristics of patients with CFN. **Table S3.** Detailed treatments modalities and outcomes of patients with CFN and renal parameters follow-up. STROBE Checklist.
