# Supplementary material for: Clinicopathological Spectrum and Treatment Outcomes of Cryofibrinogen-Associated Nephropathies
Source: Kidney Int Rep. 2025 Nov 5;11(1):129–40. doi: 10.1016/j.ekir.2025.10.022 (PMC12799572; doi:10.1016/j.ekir.2025.10.022)
Supplement: Supplementary File (PDF) — Supplementary Data. Table S1. Detailed renal clinical and biological characteristics of patients with CFN at the time of diagnosis. Table S2. Detailed renal histopathological characteristics of patients with CFN. Table S3. Detailed treatments modalities and outcomes of patients with CFN and renal parameters follow-up. STROBE Checklist. [file mmc1.pdf]

Supplemental material

Supplemental data

Patient n°19 presented with a severe glomerulonephritis with stage 3 AKI, high range proteinuria, and a nephrotic syndrome subsequently, a monoclonal IgG lambda leading to the diagnosis of a multiple myeloma (10% plasmocytosis) without evidence for an associated cast nephropathy at kidney biopsy. Treatment with CS + CYC + Bor led to the resolution of proteinuria, the improvement and stabilization of eGFR after 6 months. Patient n°20 had a severe nephrotic syndrome with stage 1 AKI and a monoclonal IgG Kappa. Treatment with CS, then CNI at 3 months, had no effect on proteinuria and led to the degradation of eGFR. Subsequent treatment with Dara + CS dramatically improved proteinuria and kidney function. Patient n°21 had a similar clinical presentation, together with a monoclonal IgG Kappa, but a treatment with CS and then Dara + Bor did not improve kidney function after 3 months. Ten sessions of PE and 4 perfusions of RTX also failed to decrease proteinuria. Finally, after 1 year, a combination of Dara + Rev + CS was started and helped normalizing proteinuria and stabilizing eGFR. Patient n°25 presented with nephrotic syndrome and stage 2 AKI, and a diagnosis of IgG Kappa multiple myeloma (10% plasmocytosis) without evidence for an associated cast nephropathy at kidney biopsy. Initial treatment with CS + Dara + Bor led to an improvement of kidney function and a partial remission of proteinuria which unfortunately increased again with recurrent lower limbs edema and uncontrolled hypertension after 5 cycles. RTX was initiated at 6 months and led to a complete remission of proteinuria. Because of a renal relapse after 18 months, RTX treatment was resumed together with Isa + CS, leading again to a complete response. On the other hand, patient n°22 had a poor response to multiple lines of treatments. He initially presented with necrotic purpura, mild AKI, non-nephrotic

proteinuria and a monoclonal IgG Kappa. Successive treatments with CS + RTX, RTX alone, then PE + CYC over one year, allowed the control of skin but not renal symptoms. He then underwent multiple lines of treatments with PE + CS + RTX, especially during winters for recurrent skin lesions, and started maintenance dialysis 10 years after the diagnosis. Patient n°27 case was peculiar because of a stage 2 AKI with mild proteinuria, a monoclonal IgA Kappa, and a classical TMA presentation with uncontrolled hypertension, and capillary but also arteriolar thrombi at kidney biopsy. There were no biological signs of hemolysis, and alternate complement pathway investigation was normal. ANA test was positive without specificity, and there were no coexisting antiphospholipid antibodies. Eculi was started alone soon after diagnosis, leading to a rapid improvement of kidney function. The treatment was stopped after 6 months, with a renal relapse, but unfortunately the patient had moved to another city and was lost to follow-up.

Table S1. Detailed renal clinical and biological characteristics of patients with CFN at the time of diagnosis

Patient	Gender[a]	Age	Suspected cause of CF	Kidney-limited CF	HBP[b]	HU	UPCR (mg/mmol)	ALB (g/L)	Baseline eGFR (mL/mn/1.73m^2)	Creatinine at diagnosis (µmol/L)	AKI staging	CF level[c]	Fg (g/L)	Complement activity	MG (g/L)	Bone marrow aspirate	CG (mg/l)
**Group 1: Essential form**																	
1	F	48	Essential	0	1	1	470	22	53	247	2	ND	6,5	Normal	0	0	0
2	M	46	Essential	1	1	1	20	40	ND	528	NA	ND	7,7	Normal	0	0	0
3	M	89	Essential	1	1	1	158	39	38	202	1	ND	4,5	Normal	0	0	0
4	M	49	Essential	0	1	1	291	28	ND	360	NA	35 mg/l	5,7	Normal	0	0	Polyclonal (31)
5	M	61	Essential	0	1	1	400	30	51	220	2	44 mg/l	5,2	Low C3/C4	0	Normal	IgMk (90)
6	M	62	Essential	0	1	0	114	44	ND	313	NA	418 mg/l	4	Normal	0	0	0
7	F	65	Essential	1	1	1	495	25	36	224	2	55 mg/l	4,7	Normal	0	0	IgGk (25)
8	M	30	Essential	0	1	1	300	31	40	296	2	217 mg/l	ND	Normal	0	0	IgMk (65)
9	F	49	Essential	1	0	0	604	44	69	321	3	29 mg/l	ND	Normal	0	0	Polyclonal (49)
**Group 2: Other secondary form**																	
10	F	71	Skin infection	0	1	1	1729	29	55	490	3	ND	6,8	Normal	0	0	Polyclonal (430)
11	M	83	Joint infection	0	0	1	500	11	41	384	3	192 mg/l	6,4	Normal	0	0	0
12	M	51	Hepatitis C	0	1	0	90	37	32	425	3	534 mg/l	ND	Normal	0	0	IgMk (442)
13	F	49	Cancer	0	0	1	194	32	ND	741	NA	ND	4,9	ND	0	0	0
14	F	57	Cancer	1	1	0	490	44	104	52	0	530 mg/l	6,7	ND	0	0	0
15[d]	M	69	Cancer	0	0	1	430	24	20	354	1	ND	12,2	Normal	0	0	0
16	F	68	Cancer	1	1	0	40	40	90	136	2	240 mg/l	5,8	Normal	0	0	0
17	M	55	Cancer	1	1	1	252	28	24	259	0	375 mg/l	7,5	Normal	0	0	Polyclonal (269)
**Group 3: Associated with Monoclonal gammopathy**																	
18	F	42	MG	0	1	1	590	38	114	55	0	6%	4,8	Normal	IgGk (1.9)	0	0
19[d]	F	52	MG	0	1	1	140	26	115	625	3	2%	9,6	Normal	IgGλ (25)	MM (Vλ3-21)	0
20	F	32	MG	0	1	1	500	12	129	71	1	623 mg/l	5,4	Normal	IgGk (0.9)	Normal	0
21	M	35	MG	0	1	1	370	28	81	108	1	ND	1,5	Low C4	IgGk (1.2)	Normal	0
22	M	69	MG	0	0	1	300	ND	ND	116	NA	4 (scale)	ND	Low C4	IgGk (NQ)	0	0
23	M	71	MG	0	1	1	360	28	49	149	0	3 (scale)	ND	Low C3	IgGk (4.9)	0	0
24	M	88	MG	0	1	1	300	39	20	257	0	ND	5,1	Normal	IgGλ (NQ)	0	0
25	M	78	MG	0	1	1	360	23	87	121	2	180 mg/l	0,9	Low C3/C4	IgGk (1.8)	MM	0
26	M	68	MG	0	0	1	386	15	103	137	2	74 mg/l	4,1	Normal	IgGλ (NQ)	Normal	Polyclonal (112)
27[d]	M	62	MG	1	0	1	99	25	63	242	2	83 mg/l	ND	Normal	IgAk (6.6)	Normal	Polyclonal (22)
28	M	78	MG	0	1	1	150	40	43	282	2	ND	7,4	Normal	IgGk (NQ)	0	0

AKI = acute kidney injury, ALB = albuminemia, CF = cryofibrinogen, CFN = cryofibrinogen-related nephropathy, CG = cryoglobulin, eGFR = estimated glomerular filtration rate according to CKD-EPI (Chronic Kidney Disease EPIdemiology collaboration), Fg = fibrinogen, HBP = high blood pressure, HU = hematuria, MG = monoclonal gammopathy, MM = multiple myeloma, NA = not applicable, ND = no data, NQ = not quantifiable, UPCR = urinary protein-to creatinine-ratio.

[a] Female (F) or Male (M). [b] HBP was defined by a blood pressure >140/90 mmHg

[c] CF levels were assessed according to the laboratory's specific protocol and given in: mg/l, percentage (%) or visual scale (from 1 to 5).

[d] Patients with past medical of diabetes mellitus

Table S2. Detailed renal histopathological characteristics of patients with CFN

	Glomeruli									Thrombi			Interstitium				
Patient	Double contours	Mesangial thickening	Mesangial nodules	FSGS	Sclerotic glomeruli	Mesangial HC	Endocapillary HC[a]	Extracapillary HC	Capillary tuft retraction	Glomerular capillaries	Arterioles	Arteries	Fibrosis [b]	Immune infiltration[c]	Fg staining	Form	Electron microscopy
**Group 1: Essential form**																	
1	0	0	0	0	0/4	1	Poly/Mono	0	0	0	1	0	2	0	+++	MPGN/TMA	
2	0	0	0	1	ND	0	0	0	0	0	1	0	2	0	0	TMA	
3	1	1	0	0	5/15	1	0	0	0	0	0	0	2	1	ND	MPGN	
4	1	0	0	0	1/16	0	Poly	0	1	0	1	0	0	0	0	MPGN/TMA	
5	1	0	0	1	2/12	0	Poly/Mono	0	0	0	0	0	1	0	0	MPGN	
6	0	0	0	1	2/7	0	0	0	1	0	1	0	2	2	0	TMA	
7	1	1	1	1	17/29	1	0	Cellular	0	0	0	0	1	0	ND	MPGN	NS deposits
8	0	0	0	0	7/12	0	0	0	1	0	1	0	3	2	0	TMA	
9	0	0	0	0	1/14	1	0	0	1	0	0	0	1	0	0	Ischemic	
**Group 2: Other secondary form**																	
10	1	1	0	0	2/14	1	Poly/Mono	0	1	0	0	0	2	0	++	MPGN	
11	0	0	0	0	9/16	0	Mono	0	1	0	0	0	2	2	0	Ischemic	
12	0	0	0	0	3/11	1	0	0	1	0	0	0	2	0	0	Ischemic	
13	0	0	0	0	0/1	0	0	0	0	1	0	0	0	0	0	TMA	
14	1	1	0	0	1/26	0	0	0	1	0	0	0	0	0	0	TMA	
15	0	1	0	0	13/21	0	0	0	1	0	0	0	3	0	ND	Ischemic	
16	0	1	0	0	5/33	0	0	0	1	0	0	0	1	0	0	Ischemic	
17	0	1	1	0	4/12	0	0	0	1	0	0	0	2	0	0	Ischemic	Non contributive
**Group 3: Associated with Monoclonal gammopathy**																	
18	1	0	0	0	1/10	0	Mono	0	0	1	0	0	0	0	++	MPGN/TMA	
19	1	0	0	1	1/20	0	Poly/Mono	Fibrocellular	1	1	0	0	3	3	+++	MPGN/TMA	NS deposits
20	1	1	1	1	1/35	1	Mono	0	0	0	0	0	1	1	++	MPGN	NS deposits
21	1	0	0	1	0/28	1	Mono	0	0	0	0	0	1	1	++	MPGN	NS deposits
22	1	ND	ND	ND	ND	ND	ND	ND	ND	1	ND	ND	ND	ND	ND	MPGN/TMA	
23	1	1	1	1	9/20	0	Poly/Mono	0	0	0	0	0	1	1	ND	MPGN	Typical CFN
24	1	1	1	0	9/36	1	Mono	0	0	0	0	0	2	1	0	MPGN	Typical CFN
25	1	1	1	1	1/14	1	Poly/Mono	Cellular	0	1	0	0	0	0	0	MPGN/TMA	
26	1	1	1	0	2/12	1	Mono	0	1	0	0	0	1	1	0	MPGN	
27	1	1	0	0	4/18	0	Poly/Mono	0	1	1	1	0	1	2	0	MPGN/TMA	TMA
28	0	1	1	0	5/19	0	Poly/Mono	0	1	1	0	1	2	0	+	TMA	

CFN = cryofibrinogen-related nephropathy, Fg = fibrinogen, FSGS = focal et segmental glomerulosclerosis, HC = hypercellularity, ND = no data, NS = non-organized subendothelial deposits.

[a] Poly = polynuclear infiltrate, mono = mononuclear infiltrate

[b] 0 = <5%, 1 = 6-25%, 2 = 26-50%, 3 = >50% of the cortical surface

[c] 0 = <10%, 1 = 10–25%, 2 = 26–50%, 3 = >50% of the non-fibrous surface

Table S3. Detailed treatments modalities and outcomes of patients with CFN and renal parameters follow-up

Patient	Follow-up after CFN diagnosis (months)	Treatment n°1	Outcomes	Treatment n°2	Outcomes	Comments
**Group 1: Essential form**						
1	6	CS 1 mg/kg/d + 6 x CYC 0.5 g/m²	RR: eGFR 19 to 26 (↗52%) + normalization of PU at 3 months	-	-	No relapse at 6 months
2	36	Nephroprotection[a]	RR: eGFR 10 to 30 (↗200%) at 3 months	-	-	Stable renal function until 36 months
3	12	Nephroprotection	No RR: eGFR 24 to 12 (↘50%) + stable PU at 3 months, dialysis at 12 months	-	-	Stroke after 12 months, severe disability and decision to limit care
4	36	CS IV 500 x 3 + Eculi 18 months	RR: eGFR 16 to 50 (↗213%) + normalization of PU at 12 months	-	-	Stable renal function until 36 months
5	24	Eculi 24 months	No RR: eGFR 27 to 23 (↘15%) + partial decrease of PU 4 to 1 g/g at 24 months	-	-	Stable renal function until 24 months
6	0	-	-	-	-	Loss to follow-up after diagnosis
7	0	CS 0.5 mg/kg/d	-	-	-	Sudden death shortly after discharge
8	36	Nephroprotection	No RR: eGFR 23 to 21 (↘9%) + partial decrease of PU 3 to 1 g/g at 36 months	-	-	Stable renal function until 36 months
9	6	Nephroprotection	No RR: eGFR 14 to 11 (↘21%) + partial decrease of PU 6 to 1 g/g at 6 months	-	-	Stable renal function until 6 months
**Group 2: Other secondary form**						
10	6	ATB + CS IV 250 x 2 then 10 mg/d, and 5 mg at 6 months	RR: eGFR 7 to 27 (↗286%), weaning from dialysis + normalization of PU at 3 months	-	-	Stable renal function until 6 months
11	0	ATB + CS 0.5 mg/kg/d 10 days	-	-	-	Loss to follow-up after diagnosis
12	36	CS IV 500 x 3 then 1 mg/kg/d, 5 mg/d at 18 months + PE x 9 + CYC 3 months stopped for AZA	RR: eGFR 13 to 18 (↗38%) + normalization of PU at 12 months	-	-	Stable renal function until 36 months<br>Start of HCV antiviral
13	0	-	-	-	-	Stroke and sudden death after diagnosis
14	0	-	-	-	-	Loss to follow-up after diagnosis
15	0	-	-	-	-	Loss to follow-up after diagnosis
16	0	-	-	-	-	Loss to follow-up after diagnosis
17	6	Nephroprotection	No RR: eGFR 23 to 21 (↘9%) + stable PU at 6 months	-	-	Stable renal function until 6 months
**Group 3: Associated with Monoclonal gammopathy**						
18	0	-	-	-	-	Loss to follow-up after diagnosis
19	36	CS + Bor + CYC (29 cycles)	RR: eGFR 6 to 40 (↗567%), weaning from dialysis + normalization of PU at 12 months	-	-	Stable renal function until 36 months
20	36	CS 1 mg/kg/d, and 5 mg/d + CNI at 3 months	No RR: eGFR 97 to 58 (↘40%) + stable PU at 18 months	6 x Dara + CS at 18 months<br>Stop CNI	RR: eGFR 58 to 102 (↗76%) + decrease of PU 5 to 1.5 g/g at month 24	Stable renal function until 36 months
21	24	CS 0.7 mg/kg/d and 5 mg/d at month + 6 x Dara + Bor at 3	No RR: eGFR 76 to 59 (↘22%) + stable PU at 12 months	6 x Dara + Rev + CS	RR: eGFR 59 to 60 (↗0%) + normalization of PU at month 24	Stable renal function at 24 months

		months, then 4 x RTX 375 mg/m² + 10 x PE at 6 months				
22	36	CS 1 mg/kg/d + 4 x RTX 375 mg/m², new cycle of 4 x RTX 375 mg/m² at 6 months	No RR: eGFR 55 to 40 (↘27%) + stagnation of PU at 6 months	5 x CYC 1g + 15 x PE at 12 months CS 1 mg/kg/d + PE + RTX/ 3 months at 24 months	No RR: eGFR 40 to 22 (↘45%) + stagnation of PU at 36 months	Progressive decline in renal function at 36 months, end-stage renal disease at 10 years
23	0	-	-	-	-	Initially misdiagnosed as C3 glomerulopathy 5 years earlier: treatment with Eculi and CT resulted in partial creatinine decrease (300 ↘ 130) but persistent nephrotic range PU. Spacing Eculi from every 2 to 4 weeks triggered multiple renal flares. Final CFN diagnosis after a repeated kidney biopsy in front of new-onset peripheral neuropathy. No prospective data after CFN diagnosis.
24	0	-	-	-	-	Loss to follow-up after diagnosis
25	24	6 x CS + Bor + Dara, then 4 x RTX 375 mg/m² at 6 months	RR: eGFR 49 to 86 (↗76%) + normalization of PU at 12 months	CS + RTX + Isa at 18 months for renal relapse (PU 2.7 g/g)	RR: eGFR 82 to 86 (↗5%) + normalization of proteinuria at 24 months	Death (infectious pneumonia)
26	36	Nephroprotection	RR: eGFR 45 to 65 (↗44%) + normalization of PU at 6 months	-	-	Stable renal function until 36 months
27	18	Eculi 6 months	RR: eGFR 24 to 40 (↗67%) + normalization of PU at 6 months	-	-	Progressive decline in renal function and recurrence of PU after Eculi withdrawal
28	0	-	-	-	-	Loss to follow-up after diagnosis

ATB = antibiotic, AZA = azathioprin, Bor = bortezomib, BP = blood pressure, CNI = calcineurin inhibitor, CS = corticosteroids, CYC = cyclophosphamide, Dara = daratumumab, Eculi = eculizumab, eGFR = estimated glomerular filtration rate according to CKD-EPI (Chronic Kidney Disease EPIdemiology collaboration), HCV = hepatitis C virus, Isa = isatuximab, PE = plasma exchange, PU = proteinuria, Rev = revlimid, RR = renal response.

[a] Nephroprotection included SRAA blockers and BP control <140/90 mmHg.

STROBE Statement—Checklist of items that should be included in reports of ***cohort studies***

	Item No	Recommendation	Page No
**Title and abstract**	1	(*a*) Indicate the study's design with a commonly used term in the title or the abstract	1
		(*b*) Provide in the abstract an informative and balanced summary of what was done and what was found	2
**Introduction**			
Background/rationale	2	Explain the scientific background and rationale for the investigation being reported	4-5
Objectives	3	State specific objectives, including any prespecified hypotheses	5
**Methods**			
Study design	4	Present key elements of study design early in the paper	6-7
Setting	5	Describe the setting, locations, and relevant dates, including periods of recruitment, exposure, follow-up, and data collection	6-7
Participants	6	(*a*) Give the eligibility criteria, and the sources and methods of selection of participants. Describe methods of follow-up	6-7
		(*b*) For matched studies, give matching criteria and number of exposed and unexposed	NA
Variables	7	Clearly define all outcomes, exposures, predictors, potential confounders, and effect modifiers. Give diagnostic criteria, if applicable	6-7
Data sources/ measurement	8*	For each variable of interest, give sources of data and details of methods of assessment (measurement). Describe comparability of assessment methods if there is more than one group	6-7
Bias	9	Describe any efforts to address potential sources of bias	6-7
Study size	10	Explain how the study size was arrived at	NA
Quantitative variables	11	Explain how quantitative variables were handled in the analyses. If applicable, describe which groupings were chosen and why	7
Statistical methods	12	(*a*) Describe all statistical methods, including those used to control for confounding	7
		(*b*) Describe any methods used to examine subgroups and interactions	7
		(*c*) Explain how missing data were addressed	7
		(*d*) If applicable, explain how loss to follow-up was addressed	7
		(*e*) Describe any sensitivity analyses	7
**Results**			
Participants	13*	(a) Report numbers of individuals at each stage of study—eg numbers potentially eligible, examined for eligibility, confirmed eligible, included in the study, completing follow-up, and analysed	8
		(b) Give reasons for non-participation at each stage	8
		(c) Consider use of a flow diagram	8
Descriptive data	14*	(a) Give characteristics of study participants (eg demographic, clinical, social) and information on exposures and potential confounders	8-9
		(b) Indicate number of participants with missing data for each variable of interest	8-9
		(c) Summarise follow-up time (eg, average and total amount)	9
Outcome data	15*	Report numbers of outcome events or summary measures over time	9-10

Main results	16	(*a*) Give unadjusted estimates and, if applicable, confounder-adjusted estimates and their precision (eg, 95% confidence interval). Make clear which confounders were adjusted for and why they were included<br>(*b*) Report category boundaries when continuous variables were categorized<br>(*c*) If relevant, consider translating estimates of relative risk into absolute risk for a meaningful time period	9-11<br>9-11<br>NA
Other analyses	17	Report other analyses done—eg analyses of subgroups and interactions, and sensitivity analyses	NA
**Discussion**			
Key results	18	Summarise key results with reference to study objectives	11-17
Limitations	19	Discuss limitations of the study, taking into account sources of potential bias or imprecision. Discuss both direction and magnitude of any potential bias	16-17
Interpretation	20	Give a cautious overall interpretation of results considering objectives, limitations, multiplicity of analyses, results from similar studies, and other relevant evidence	11-17
Generalisability	21	Discuss the generalisability (external validity) of the study results	16-17
**Other information**			
Funding	22	Give the source of funding and the role of the funders for the present study and, if applicable, for the original study on which the present article is based	17

*Give information separately for exposed and unexposed groups.

Note: An Explanation and Elaboration article discusses each checklist item and gives methodological background and published examples of transparent reporting. The STROBE checklist is best used in conjunction with this article (freely available on the Web sites of PLoS Medicine at http://www.plosmedicine.org/, Annals of Internal Medicine at http://www.annals.org/, and Epidemiology at http://www.epidem.com/). Information on the STROBE Initiative is available at http://www.strobe-statement.org.
